# Book Review: Cosmeceuticals and Active Cosmetics, Third Edition

**DOI:** 10.3389/fphar.2019.00529

**Published:** 2019-05-24

**Authors:** Marco Nuno De Canha, Analike Blom van Staden, Bianca Daphne Fibrich, Isa Anina Lambrechts, Lilitha Denga, Namrita Lall

**Affiliations:** ^1^Department of Plant and Soil Sciences, University of Pretoria, Pretoria, South Africa; ^2^School of Natural Resources, University of Missouri, Columbia, MO, United States; ^3^College of Pharmacy, JSS Academy of Higher Education and Research, Mysuru, India

**Keywords:** cosmeceuticals, cosmetics, skin care, hair care, natural ingredients

Cosmeceuticals are ingredients or products that possess cosmetic and pharmaceutical benefits, which can be obtained without a prescription (Sharad, 2019). This book (Sivamani et al., 2015) provides the reader with ample information regarding ingredients and commercial products which hail largely from botanical origin, but also includes a chapter on biomarine active ingredients. It not only illustrates the diverse applications of cosmeceutical ingredients in skin, hair, and nail care but also their pharmaceutical indications. The causes and potential mechanisms for the development and progression of several skin conditions and their therapeutic targets are also described.

A general overview of phytochemicals/botanical extracts and their use in skin care is provided in chapters 1, 2, 3, 7, and 10. This includes how certain phytochemicals such as bakuchiol contain functional groups such as terpenic moieties which offer enhanced efficacy when compared to mainstream compounds such as retinol (chapter 1). Compounds such as caffeine which are consumed regularly, offer relief to a plethora of skin conditions when applied topically, with the added benefit of enhanced skin penetration (chapter 2). Chapter 3 delves into the potential of curcumin for the treatment of photocarcinogenesis, validated by clinical trials. The use of hexylrescorinol as an anesthetic in throat lozenges and several additional molecular targets is divulged in chapter 7, while chapter 10 provides a general overview on the use of resveratrol-rich grape skins and seeds and their copious incorporation into commercial products.

Pigmentary aberrations as a function of disorders such as vitiligo or UV-induced hyperpigmentation are discussed in chapters 5, 27, and 29 (Njoo and Westerhof, 2001). Chapter 5 specifically discusses the use of ellagic acid from pomegranate as an agent capable of rectifying UV-induced hyperpigmentation comparable to the efficacy of arbutin.

The pathophysiology of several diseases associated with impaired barrier function and dehydrated skin are elaborated in chapters 19 and 26, which highlight the importance of moisturizers and emollients and the respective mechanisms by which they are able to relieve these conditions. The efficacy of fatty acids such as gamma linoleic acid found in the oils of the genera *Borago, Oenothera*, and *Echium* (chapter 6) and amino acids (chapter 15) as wound healing and skin hydration agents is also covered.

Chapter 21, 22, and 23 emphasized how skin homeostasis is disturbed by exposure to UV rays, which may manifest as premature aging and skin cancer. These chapters, along with chapter 16 critically discussed the benefits of topical antioxidants in neutralizing reactive oxygen species and how they can be applied individually or in combination, depending on their chemical properties and clinical efficacy. Furthermore, chapter 8, 9, 11, 12, 13, and 14 discusses the use of plant hormones (cytokinins) with high skin penetration and low irritancy as DNA repair agents for aging skin and phenylpropanoids from *Rhodilia rosea*, silymarin from milk thistle, niacinamide, anti-aging peptides and proteins in the prevention of photodamage. Peptides can be carriers of anti-aging ingredients, inhibitors of enzymes contributing to aging or act as signals or neurotransmitters to reverse factors contributing to skin damage. The use of hydroxyacids (both α and β), commonly known as fruit acids as common ingredients in skin peeling products and their and potential adverse effects (stinging, irritation, swelling and redness of the skin) is also discussed in chapter 8.

Chapter 28 focused on the cosmeceutical treatment of disorders associated with sebaceous glands and the need for more clinical data while chapter 24 explored the use of botanical extracts such as the feverfew tree, licorice, *Crysanthellum indicum* and *Quassia amara* for rosacea as independent or complementary adjuvant therapies.

This book highlights the potential of phytoconstituents to maintain health across the board but also highlights that the most hindering factor in the development and commercialization of natural products is a lack of efficacy studies in human trials. Even in instances where abundant phytochemicals such as epicatechin-3-gallate which comprises 50–80% of green tea provide sufficient and even surplus information in cell culture and animal studies, the incorporation into cosme- and pharmaceuticals for human consumption is limited (chapter 4). The exploration of marine organisms as a source of active compounds such as fucoidan from brown seaweed may provide a plethora of novel natural actives from which new medicines can be developed (chapter 31).

Non-skin related conditions are discussed in chapter 18, 20, and 25, which includes hair and nail care. Chapter 18 considers hair structure and function and how factors such as hydrophobicity, heat, oil content and the degree of damage govern the ingredients that need to be used in shampoos, conditioners and fixatives. Chapter 25 discusses condition known as androgenic alopecia, which is characterized by hair loss. Various extracts and cosmeceutical compounds involved in hormone and hormone receptor balance are described. Treatments for unhealthy nails and various nail conditions are discussed in chapter 20.

Important concepts in the manufacturing of natural actives including the influence of extraction processes on bioactivity against several conditions are highlighted in chapter 31, while chapter 33 discusses the use of techniques in the quantification of bioactive compounds from extracts.

Preservatives, antioxidants and natural pigments can also provide crucial functions to the integrity of a formulation as covered in chapters 17, 19, and 26. The use of preservatives and anti-oxidants to prevent bacterial growth and browning in moisturizers is a noteworthy section discussed in chapter 19 and 26. Chapter 17 describes natural pigments, both organic and inorganic in decorative cosmetics like make-up (Parish and Crissey, 1988). Perhaps the highlight of this section in terms of cosmeceutical use, was nano dispersion forms of administration of TiO_2_ and ZnO for UVB protection.

Finally, chapter 34 identified legal terms as outlined by the Federal Food, Drug, and Cosmetic Act and what constitutes an agent as a drug or cosmetic which is becoming an increasingly controversial topic.

Although the book provides the reader with extensive scientific data relating to the efficacy of natural products, this limits the readership to researchers in particular fields, requiring some knowledge of associated biological pathways. While the book discusses validation of efficacy, perhaps more focus needs to be placed on other important features of cosmeceuticals, including safety and potential interactions with other products. The quality of the book could also be improved by the inclusion of specific mechanisms of action in addition to the antioxidant data, which has become somewhat over-emphasized in natural product research. The reputation of natural product actives would be valued a great deal more if multiple, concrete mechanisms of action are identified for treating a disorder. Lastly, although a vast amount of information has been superbly summarized in this book, future reviews should perhaps focus on current trends in cosmeceutical and pharmaceutical research that have not been so extensively covered. Cosmeceutical research has entered an era where repeating tedious experiments on new leads, no longer constitutes cutting edge or interesting research. Researchers are more inclined to find alternative methods that enhance the frugality of existing medicinal extracts through a multidisciplinary approach to generate not only revolutionary science, but products too.

## Author Contributions

All authors listed have made a substantial, direct and intellectual contribution to the work, and approved it for publication.

### Conflict of Interest Statement

The authors declare that the research was conducted in the absence of any commercial or financial relationships that could be construed as a potential conflict of interest.
